# Peer review of "Three-Dimensional Imaging of Circular Array Synthetic Aperture Sonar for Unmanned Surface Vehicle"

_sensors, 2022, doi:10.3390/s22103797_

Round 1

Reviewer 1 Report

In this paper, the authors have looked at a 3D image reconstruction technique utilizing sparse Bayesian learning, for the application of three-dimensional accurate imaging for underwater target, which is benefit for verification and identification of underwater target. In general, the authors have presented the methods well and the mathematical analysis are clear. They have also clearly demonstrated their experimental setup and their simulation results are convincing.

My major concern is regarding the English usage, which needs to be edited well before the final submission.

Some language issues spotted:

In section 3.2: "In order to increase the array size of CASAS, which is benefit for the resolution in
the height direction. The dense of CASAS line array cannot satisfy the half-wavelength
spacing condition, which cannot avoid the grating lobes." These sentences does not look complete on their own.

In the conclusions: "For the application of three-dimensional accurate imaging for underwater target, which is benefit for verification and identification of underwater target." This looks an incomplete sentence. 

Author Response

Dear reviewer,

    Thank you very much for your comments and suggestions. My responses to the comments can be seen as follow.

(1) Your comments: “In this paper, the authors have looked at a 3D image reconstruction technique utilizing sparse Bayesian learning, for the application of three-dimensional accurate imaging for underwater target, which is benefit for verification and identification of underwater target. In general, the authors have presented the methods well and the mathematical analysis are clear. They have also clearly demonstrated their experimental setup and their simulation results are convincing.”

  My responses: Thanks very much for your comments.

(2) Your comments:“My major concern is regarding the English usage, which needs to be edited well before the final submission.

Some language issues spotted:

In section 3.2: "In order to increase the array size of CASAS, which is benefit for the resolution in the height direction. The dense of CASAS line array cannot satisfy the half-wavelength spacing condition, which cannot avoid the grating lobes." These sentences does not look complete on their own.

In the conclusions: "For the application of three-dimensional accurate imaging for underwater target, which is benefit for verification and identification of underwater target." This looks an incomplete sentence.

My responses: Thanks very much for your suggestions. Before submitting the final draft, I have carefully checked the English grammar and expression of the article.

About the language issues you spotted:

1) In section 3.2: "In order to increase the array size of CASAS, which is benefit for the resolution in the height direction. The dense of CASAS line array cannot satisfy the half-wavelength spacing condition, which cannot avoid the grating lobes." These sentences does not look complete on their own.

  The author agrees with your comment that the sentences are really incomplete. The modified sentences can be seen as follows:

“In order to increase the array size of CASAS, which is benefit for the resolution in the height direction, the sparse hydrophone sensors array along the Z-axis is implemented. The dense of CASAS line array cannot satisfy the half-wavelength spacing condition, which cannot avoid the grating lobes.”

2) In the conclusions: "For the application of three-dimensional accurate imaging for underwater target, which is benefit for verification and identification of underwater target." This looks an incomplete sentence.

 The author agrees with your comment that the sentences are really incomplete. The modified sentences can be seen as follows:

“Three-dimensional imaging is benefit for verification and identification of underwater target. For the application of 3D accurate imaging for underwater target, a novel 3D imagine process combines CASAS with multiple receivers was proposed in this study.”

(3) For other modifications of this paper, please refer to the revised draft. The main modifications are described as follows:

  • In section 1, line 29~30: “Synthetic aperture sonar (SAS) is a well-established powerful underwater remote-sensing technique for generating imagery from scenes that have been insonified by a sonar platform moving in a trajectory [1,2,3,4]. SAS systems could transmit acoustic pulses while moving along a trajectory and coherently combine the backscattered echoes to yield high-resolution images of the objects [5]” >” Synthetic aperture sonar (SAS) is a well-established powerful underwater remote-sensing technique [1,2,3,4]. SAS systems could transmit acoustic pulses while moving along a trajectory and coherently combine the backscattered echoes to yield high-resolution images of the objects [5,6]”
  • In section 1, line 73~90:” However, the synthesized multipass array will be irregular of AUV-mounted sonar system due to navigation error which caused by currents [21,22]. This matter can fail to coherently combine the backscattered signals, which lead to cannot obtain 3D imaging of target. On the other hand, the AUV-mounted sonar system need to rise to the surface frequently for GPS localization to corrected underwater navigation trajectory during multipass measurement, which is time-consuming. This paper proposes a novel method for overcoming these difficulties by demonstrations of CSAS tomography from circular array synthetic aperture sonar(CASAS) mounted in unmanned surface vehicle(USV). The structure of this paper is as follows.” >” However, the synthesized multipass array will be irregular of AUV-mounted sonar system due to navigation error which caused by currents [24,25,26]. This problem can fail to coherently combine the backscattered signals, which lead to obtain 3D imaging of target unsuccessfully . On the other hand, the AUV-mounted sonar system would surface and get a new GPS lock frequently for correcting underwater navigation trajectory during multipass measurement [25], which is time-consuming, what’s more, error in the GPS localization would often cause a discontinuity in the final multipass aperture. This paper proposes a novel method to overcome these difficulties, it uses Unman Surface Vehicle (USV)-mounted array in height direction sonar system insonifies targets using a single circular scan, then coherently combine the backscattered signals to obtain 3D imaging of target. Similar to the definition of Circular Array SAR (CASAR) in literature [27], the USV-mounted array in height direction can be defined as Circular array synthetic aperture sonar (CASAS). The structure of this paper is organized as follows. In section 2, the CASAS system configuration, parameters, signal model and data acquisition are introduced. In section 3, the signal calibration, scattering coefficient reconstruction along the height direction, 3D imaging reconstruction procedure and point spread function of CASAS are presented. The corresponding simulation and experimental results are shown in section 4. In section 5, it concludes the paper.”
  • In section 3.2, line 235~236:” In order to increase the array size of CASAS, which is benefit for the resolution in the height direction. The dense of CASAS line array cannot satisfy the half-wavelength spacing condition, which cannot avoid the grating lobes.” ━> “In order to increase the array size of CASAS, which is benefit for the resolution in the height direction, the sparse hydrophone sensors array along the Z-axis is implemented. The dense of CASAS line array cannot satisfy the half-wavelength spacing condition, which cannot avoid the grating lobes.”
  • In section 5, line 458~459:” For the application of three-dimensional accurate imaging for underwater target, which is benefit for verification and identification of underwater target. A novel 3D imagine process combines CASAS with multiple receivers was proposed in this study.” ━> “Three-dimensional imaging is benefit for verification and identification of underwater target [43,44,45]. For the application of 3D accurate imaging for underwater target, a novel 3D imagine process combines CASAS with multiple receivers was proposed in this study.”
  • In section references, according to the suggestions of the reviewers, the author added some new references.

Reviewer 2 Report

The submission describes work on Circular Synthetic Aperture Sonar (CSAS). The work is in principle interesting and there are especially some interesting results from real world experiments. But it clearly also needs some polishing in terms of the presentation. This includes especially the language. I am not a language speaker myself and there are quite many glitches; I hence refrain from listing them all and from making suggestions regarding the language.

Also, the abbreviation CASAS that is extentively used throughout the submission is not defined. I simply assume it is meant to stand for Circular Array Synthetic Aperture Sonar. Why not using the more standard term Circular Synthetic Aperture Sonar (CSAS), which is also used in the submission? If there is a specific aspect to be highlighted, it should be motivated and discussed.

The discussion of the state of the art should include some more papers on CSAS; examples that come to my mind are [A1-A3]. Also, [A4] is of interest as it describes a navigation-free extension of CSAS.

It may be an option to also mention that there is some quite active research on 3D-sonars, e.g., [B1-B9]  as a kind of alternative for 3D underwater perception - but with more complex devices. But that is as mentioned just an option.

Concluding, the work has merits. But the presentation of the submission, including especially language, must be improved.       

[A1] T. Marston, J. Kennedy, and P. Marston, “Coherent and semi-coherent processing of limited-aperture circular synthetic aperture (CSAS) data,” in Oceans 2011, Hawai, Sep 2011, pp. 1–6.

[A2] H. Tran, B. Nguyen, R. Melino, and S. Wood, “A detailed study of sonar tomographic imaging,” Maritime Operations Division Electronic Warfare and Radar Division: Research Report AR-015-647, 2013.

[A3] H. Callow, R. Hansen, S. Synnes, and T. Saebo, “Circular synthetic aperture sonar without a beacon,” in Proceedings of the 3rd international Conference & Exhibition on Underwater Acoustic Measurements, 2009.

[A4] H. Bülow and A. Birk, "Synthetic Aperture Sonar (SAS) without Navigation: Scan Registration as Basis for Near Field Synthetic Imaging in 2D," Sensors, vol. 20, 2020.

optional: 3D sonars 

[B1] D. Zhao, X. Liu, F. Zhou, R. Jiang, Y. Hu, and Y. Chen, “An efficient near-field parallel subarray beamforming for portable 3d imaging sonar,” in IEEE 3rd Advanced Information Technology, Electronic and Automation Control Conference (IAEAC), Conference Proceedings, pp. 1120–1124. 2016

[B2] M. Ehrhardt, C. Degel, F. J. Becker, L. Peter, H. Hewener, H. Fonfara, M. Fournelle, and S. Tretbar, “Comparison of different short-range sonar systems on real structures and objects,” in IEEE/MTS OCEANS. IEEE, Conference Proceedings, pp. 1–6. 2017

[B3] X. Liu, F. Zhou, H. Zhou, X. Tian, R. Jiang, and Y. Chen, “A low-complexity real-time 3-d sonar imaging system with a cross array,” IEEE Journal of Oceanic Engineering, vol. 41, no. 2, pp. 262–273, 2016.

[B4] M. Palmese and A. Trucco, “Pruned chirp zeta transform beamforming for 3-d imaging with sparse planar arrays,” IEEE Journal of Oceanic Engineering, vol. 39, no. 2, pp. 206–211, 2014.

[B5] C. Degel, H. Fonfara, H. J. Welsch, F. J. Becker, H. Hewener, M. Fournelle, and S. H. Tretbar, “3d sonar system based on mills cross antenna configuration,” in IEEE/MTS Oceans. IEEE, Conference Proceedings, pp. 1–6. 2014

[B6] T. Josserand and J. Wolley, “A miniature high resolution 3-d imaging sonar,” Ultrasonics, vol. 51, no. 3, pp. 275–280, 2011. 

[B7] P. Chen, W. Zhu, and Y. Zheng, “Research of multi-fpga signal processor for underwater 3-d imaging sonar system,” in IEEE International Conference on Electrical and Control Engineering. IEEE, Conference Proceedings, pp. 4666–4669, 2011

[B8] A. Trucco, M. Palmese, and S. Repetto, “Devising an affordable sonar system for underwater 3-d vision,” IEEE Transactions on Instrumentation and Measurement, vol. 57, no. 10, pp. 2348–2354, 2008.

[B9] V. Murino and A. Trucco, “Three-dimensional image generation and processing in underwater acoustic vision,” Proceedings of the IEEE, vol. 88, no. 12, pp. 1903–1948, 2000. 

Author Response

Dear reviewer,

    Thank you very much for your comments and suggestions. My responses to the comments can be seen as follow.

(1) Your comments: “The submission describes work on Circular Synthetic Aperture Sonar (CSAS). The work is in principle interesting and there are especially some interesting results from real world experiments. But it clearly also needs some polishing in terms of the presentation. This includes especially the language. I am not a language speaker myself and there are quite many glitches; I hence refrain from listing them all and from making suggestions regarding the language.

  My responses: Thanks very much for your comments. There are indeed some grammatical or English mistakes in the article.  The author has carefully checked and revised the manuscript, you can see the revised manuscript for details.  

(2) Your comments: “Also, the abbreviation CASAS that is extentively used throughout the submission is not defined. I simply assume it is meant to stand for Circular Array Synthetic Aperture Sonar. Why not using the more standard term Circular Synthetic Aperture Sonar (CSAS), which is also used in the submission? If there is a specific aspect to be highlighted, it should be motivated and discussed.”

  My responses: Thanks very much for your comments and suggestions. The abbreviation CASAS definition refers to the definition of Circular Array SAR (CASAR) in literature [27][Ming Jing, Zhang Xiaoling, Pu Ling, et al.. PSF analysis and ground test results of a novel circular array 3-D SAR system[J]. Journal of Radars, 2018, 7(6): 770–776. DOI: 10.12000/JR18068.]. As you described, it is meant to stand for Circular Array Synthetic Aperture Sonar. As described in the Introduction section, the standard term Circular Synthetic Aperture Sonar is mounted in AUV that insonifies targets using circular scans that vary in height, the synthesized multipass array will be irregular of AUV-mounted sonar system due to navigation error which caused by currents. In this paper, we use an array which was mounted in USV in height direction to replace the the synthesized multipass array, this method can save measuring time and is beneficial to motion compensation. In section 2, we have discussed the CASAS configuration, parameters and signal model. I hope my reply can answer your questions.

(3) Your comments: “The discussion of the state of the art should include some more papers on CSAS; examples that come to my mind are [A1-A3]. Also, [A4] is of interest as it describes a navigation-free extension of CSAS.

It may be an option to also mention that there is some quite active research on 3D-sonars, e.g., [B1-B9]  as a kind of alternative for 3D underwater perception - but with more complex devices. But that is as mentioned just an option.”

  My responses: Thanks very much for your comments and suggestions. Some papers you list have been included in the discussion of the state of the art.  You can see the revised manuscript for details.

(4) Your comments: “Concluding, the work has merits. But the presentation of the submission, including especially language, must be improved.”

  My responses: Thanks very much for your comments and suggestions. There are indeed some grammatical or English mistakes in the article.  The author has carefully checked and revised the manuscript, you can see the revised manuscript for details. The specific modifications of this paper are as follows:

  • In section 1, line 29~30: “Synthetic aperture sonar (SAS) is a well-established powerful underwater remote-sensing technique for generating imagery from scenes that have been insonified by a sonar platform moving in a trajectory [1,2,3,4]. SAS systems could transmit acoustic pulses while moving along a trajectory and coherently combine the backscattered echoes to yield high-resolution images of the objects [5]” >” Synthetic aperture sonar (SAS) is a well-established powerful underwater remote-sensing technique [1,2,3,4]. SAS systems could transmit acoustic pulses while moving along a trajectory and coherently combine the backscattered echoes to yield high-resolution images of the objects [5,6]”
  • In section 1, line 73~90:” However, the synthesized multipass array will be irregular of AUV-mounted sonar system due to navigation error which caused by currents [21,22]. This matter can fail to coherently combine the backscattered signals, which lead to cannot obtain 3D imaging of target. On the other hand, the AUV-mounted sonar system need to rise to the surface frequently for GPS localization to corrected underwater navigation trajectory during multipass measurement, which is time-consuming. This paper proposes a novel method for overcoming these difficulties by demonstrations of CSAS tomography from circular array synthetic aperture sonar(CASAS) mounted in unmanned surface vehicle(USV). The structure of this paper is as follows.” >” However, the synthesized multipass array will be irregular of AUV-mounted sonar system due to navigation error which caused by currents [24,25,26]. This problem can fail to coherently combine the backscattered signals, which lead to obtain 3D imaging of target unsuccessfully . On the other hand, the AUV-mounted sonar system would surface and get a new GPS lock frequently for correcting underwater navigation trajectory during multipass measurement [25], which is time-consuming, what’s more, error in the GPS localization would often cause a discontinuity in the final multipass aperture. This paper proposes a novel method to overcome these difficulties, it uses Unman Surface Vehicle (USV)-mounted array in height direction sonar system insonifies targets using a single circular scan, then coherently combine the backscattered signals to obtain 3D imaging of target. Similar to the definition of Circular Array SAR (CASAR) in literature [27], the USV-mounted array in height direction can be defined as Circular array synthetic aperture sonar (CASAS). The structure of this paper is organized as follows. In section 2, the CASAS system configuration, parameters, signal model and data acquisition are introduced. In section 3, the signal calibration, scattering coefficient reconstruction along the height direction, 3D imaging reconstruction procedure and point spread function of CASAS are presented. The corresponding simulation and experimental results are shown in section 4. In section 5, it concludes the paper.”
  • In section 3.2, line 235~236:” In order to increase the array size of CASAS, which is benefit for the resolution in the height direction. The dense of CASAS line array cannot satisfy the half-wavelength spacing condition, which cannot avoid the grating lobes.” ━> “In order to increase the array size of CASAS, which is benefit for the resolution in the height direction, the sparse hydrophone sensors array along the Z-axis is implemented. The dense of CASAS line array cannot satisfy the half-wavelength spacing condition, which cannot avoid the grating lobes.”
  • In section 5, line 458~459:” For the application of three-dimensional accurate imaging for underwater target, which is benefit for verification and identification of underwater target. A novel 3D imagine process combines CASAS with multiple receivers was proposed in this study.” ━> “Three-dimensional imaging is benefit for verification and identification of underwater target [43,44,45]. For the application of 3D accurate imaging for underwater target, a novel 3D imagine process combines CASAS with multiple receivers was proposed in this study.”
  • In section references, according to the suggestions of the reviewers, the author added some new references.

Reviewer 3 Report

  1. The authors derive the circular array synthetic aperture sonar based on the equivalent phase center developed for unmanned surface vehicles, and corresponding to the echo signal model, a novel three-dimensional imaging algorithm.
  2. Figure 3, shows a detailed flowchart illustrating the design concepts of the 3D imaging of circular array synthetic aperture sonar design.
  3. Please revise and enhance the English writing to improve the manuscript’s readability.

Author Response

Dear reviewer,

    Thank you very much for your comments and suggestions. My responses to the comments can be seen as follow.

(1) Your comments: “The authors derive the circular array synthetic aperture sonar based on the equivalent phase center developed for unmanned surface vehicles, and corresponding to the echo signal model, a novel three-dimensional imaging algorithm.”

  My responses: Thanks very much for your comments.

(2) Your comments: “Figure 3, shows a detailed flowchart illustrating the design concepts of the 3D imaging of circular array synthetic aperture sonar design.”

My responses: Thanks very much for your comments.

(3) Your comments: “Please revise and enhance the English writing to improve the manuscript’s readability.

My responses: Thanks very much for your comments. For other modifications of this paper, please refer to the revised draft. The specific modifications are described as follows:

  • In section 1, line 29~30: “Synthetic aperture sonar (SAS) is a well-established powerful underwater remote-sensing technique for generating imagery from scenes that have been insonified by a sonar platform moving in a trajectory [1,2,3,4]. SAS systems could transmit acoustic pulses while moving along a trajectory and coherently combine the backscattered echoes to yield high-resolution images of the objects [5]” >” Synthetic aperture sonar (SAS) is a well-established powerful underwater remote-sensing technique [1,2,3,4]. SAS systems could transmit acoustic pulses while moving along a trajectory and coherently combine the backscattered echoes to yield high-resolution images of the objects [5,6]”
  • In section 1, line 73~90:” However, the synthesized multipass array will be irregular of AUV-mounted sonar system due to navigation error which caused by currents [21,22]. This matter can fail to coherently combine the backscattered signals, which lead to cannot obtain 3D imaging of target. On the other hand, the AUV-mounted sonar system need to rise to the surface frequently for GPS localization to corrected underwater navigation trajectory during multipass measurement, which is time-consuming. This paper proposes a novel method for overcoming these difficulties by demonstrations of CSAS tomography from circular array synthetic aperture sonar(CASAS) mounted in unmanned surface vehicle(USV). The structure of this paper is as follows.” >” However, the synthesized multipass array will be irregular of AUV-mounted sonar system due to navigation error which caused by currents [24,25,26]. This problem can fail to coherently combine the backscattered signals, which lead to obtain 3D imaging of target unsuccessfully . On the other hand, the AUV-mounted sonar system would surface and get a new GPS lock frequently for correcting underwater navigation trajectory during multipass measurement [25], which is time-consuming, what’s more, error in the GPS localization would often cause a discontinuity in the final multipass aperture. This paper proposes a novel method to overcome these difficulties, it uses Unman Surface Vehicle (USV)-mounted array in height direction sonar system insonifies targets using a single circular scan, then coherently combine the backscattered signals to obtain 3D imaging of target. Similar to the definition of Circular Array SAR (CASAR) in literature [27], the USV-mounted array in height direction can be defined as Circular array synthetic aperture sonar (CASAS). The structure of this paper is organized as follows. In section 2, the CASAS system configuration, parameters, signal model and data acquisition are introduced. In section 3, the signal calibration, scattering coefficient reconstruction along the height direction, 3D imaging reconstruction procedure and point spread function of CASAS are presented. The corresponding simulation and experimental results are shown in section 4. In section 5, it concludes the paper.”
  • In section 3.2, line 235~236:” In order to increase the array size of CASAS, which is benefit for the resolution in the height direction. The dense of CASAS line array cannot satisfy the half-wavelength spacing condition, which cannot avoid the grating lobes.” ━> “In order to increase the array size of CASAS, which is benefit for the resolution in the height direction, the sparse hydrophone sensors array along the Z-axis is implemented. The dense of CASAS line array cannot satisfy the half-wavelength spacing condition, which cannot avoid the grating lobes.”
  • In section 5, line 458~459:” For the application of three-dimensional accurate imaging for underwater target, which is benefit for verification and identification of underwater target. A novel 3D imagine process combines CASAS with multiple receivers was proposed in this study.” ━> “Three-dimensional imaging is benefit for verification and identification of underwater target [43,44,45]. For the application of 3D accurate imaging for underwater target, a novel 3D imagine process combines CASAS with multiple receivers was proposed in this study.”

In section references, according to the suggestions of the reviewers, the author added some new references.

Round 2

Reviewer 2 Report

The presentation has been improved and it is now much clearer what has been actually done. My according comments are all resolved. Nonetheless, a polishing of the language is still recommended to be done for a final version.

Author Response

Dear reviewer,

    Thank you very much for your comments and suggestions. My responses to the comments can be seen as follow.

(1) Your comments: “The presentation has been improved and it is now much clearer what has been actually done. My according comments are all resolved. Nonetheless, a polishing of the language is still recommended to be done for a final version.

  My responses:

Thanks very much for your comments. Your suggestions are crucial to improving this article. There are indeed some grammatical or English mistakes in the article. The author has repeatedly revised and corrected the mistakes in the paper, and asked native English speaking colleagues to polish the language of this paper. 

The author has carefully checked and revised the manuscript, you can see the revised manuscript for details.

  The specific modifications of this paper are as follows:

  • In Abstract, line 20~22: “After the reconstruction of all height slice cells, the final 3D image was obtained. Numerical simulations and experiments using CASAS mount on USV were performed.” >” After the reconstruction of all height slice cells was accomplished, the final 3D image was obtained. Numerical simulations and experiments using USV-mounted CASAS system were performed.”
  • In section 2.1, line 114:” Considering the assumption that the target is in the far-field.” >” Considering the target is in the far-field.”
  • In section 2.2, line 118:” The pulsed linear frequency modulated (LFM) using the dechrip-on-receive technique is appropriate for CASAS imaging.” ━> “The pulsed linear frequency modulated (LFM) is appropriate for CASAS imaging..”
  • In section 2.3, line 133:” The CASAS transmission LFM signal p(τ) and the echo signal is .” ━> “The CASAS transmission signal p(τ) is the LFM signal and the echo signal is .”
  • In section4, line 273: “For CASAS imaging system, it is difficult to acquire the PSF to analyze the expression of CASAS out of the image scene center.” ━>” For CASAS imaging system, it is difficult to acquire the analyze expression of CASAS PSF out of the image scene center.”
  • In figure7, line 325: “Figure 7. The 3D imaging results circular array synthetic aperture sonar. (a) the reconstruction in height direction at angle 15o; (b) the 2D imaging result of horizontal slice at height z =-4;(c) the 3D imaging result of the simulated target.”━>” The 3D imaging simulation results of circular array synthetic aperture sonar. (a) the reconstruction in the height direction at angle 15o; (b) the 2D imaging result of horizontal slice at height z =-4;(c) the 3D imaging result of the simulated target.
  • In figure 8, line 353: “Figure 8. Experimental scenario and setup of circular array synthetic aperture sonar. (a)the schematic of CASAS installation in USV, it also shows the Navigation devices of USV, such as RTK GPS and INS. (b) the photo shows that the CASAS has been installed in USV, the transmit and receive array side of CASAS perpendicular to the move direction of USV during the trial. (c)the photo of the steel cube which was treated as experiment target. (d)the trajectory of USV in experiment by GPS.”━>” Figure 8. Experimental scenario and setup of circular array synthetic aperture sonar. (a)the schematic of CASAS installation in USV, it also shows the Navigation devices of USV, such as RTK GPS and INS. (b) the photo shows that the CASAS was installed in USV, the transmit and receive array side of CASAS perpendicular to the movement direction of USV during the trial. (c)the photo of the steel cube which was treated as experiment target. (d)the trajectory of USV in experiment which was recorded by GPS.
